# Bioactive compounds and biomedical applications of endophytic fungi: a recent review

**DOI:** 10.1186/s12934-023-02118-x

**Published:** 2023-06-06

**Authors:** Amr H. Hashem, Mohamed S. Attia, Esalm K. Kandil, Mahmoud M. Fawzi, Ahmed S. Abdelrahman, Mohamed S. Khader, Mohamed A. Khodaira, Abdallah E. Emam, Mohamed A. Goma, Amer M. Abdelaziz

**Affiliations:** Botany and Microbiology Department, Faculty of Science, Al-Azhar University, Cairo, Egypt

**Keywords:** Fungal endophytes, Medicinal plants, Antimicrobial activity, Antioxidant activity, Antiviral activity, Anticancer activity

## Abstract

Human life has been significantly impacted by the creation and spread of novel species of antibiotic-resistant bacteria and virus strains that are difficult to manage. Scientists and researchers have recently been motivated to seek out alternatives and other sources of safe and ecologically friendly active chemicals that have a powerful and effective effect against a wide variety of pathogenic bacteria as a result of all these hazards and problems. In this review, endophytic fungi and their bioactive compounds and biomedical applications were discussed. Endophytes, a new category of microbial source that can produce a variety of biological components, have major values for study and broad prospects for development. Recently, endophytic fungi have received much attention as a source for new bioactive compounds. In addition, the variety of natural active compounds generated by endophytes is due to the close biological relationship between endophytes and their host plants. The bioactive compounds separated from endophytes are usually classified as steroids, xanthones, terpenoids, isocoumarins, phenols, tetralones, benzopyranones and enniatines. Moreover, this review discusses enhancement methods of secondary metabolites production by fungal endophytes which include optimization methods, co-culture method, chemical epigenetic modification and molecular-based approaches. Furthermore, this review deals with different medical applications of bioactive compounds such as antimicrobial, antiviral, antioxidant and anticancer activities in the last 3 years.

## Introduction

Endophytes are organisms exist in living healthy plant tissues without causing disease symptoms in their host plants [1]. Through colonization sites, nutritional competition with pathogens, antibiotic synthesis, and induction of resistance mechanisms, endophytic fungi protect plants from disease [2]. Endophytic fungi may promote the growth of their host plant by producing phytohormones or by increasing the plant's resistance to various stresses, and they can produce pesticides to protect plants from herbivores [3–6]. Plants can produce vast reservoirs of interconnected microorganisms, such as bacteria and fungi, known as plant-micro biomes [7]. Endophytic fungi are a significant and hyperdiverse type of endophytes, with an estimated one million distinct fungal taxa [8, 9].

Recently, scientists have received much attention about isolation of endophytic fungi and the study of their natural products. Endophytic fungi advance to be more metabolically trends compared to soil fungi [10] or fungal associated with cyanobacteria [8]. Endophytic fungi are considered a strong source of new bioactive compounds [11]. There are many active and biologically active substances produced grouped into different categories due to the relationship between endophytes and their hosts [12]. Secondary metabolites of endophytic fungi include steroids, alkaloids, phenols, isocoumarins, xanthones, quinones, and terpenoids [13]. These fungi possess the ability to produce a large number of chemically different secondary metabolites [14], these substances are known to have antimicrobial, antifungal, antiparasitic, anticancer and antiviral activities [15, 16]. Numerous studies have reported novel, beneficial bioactive compounds exhibiting biological properties such as antibacterial, antidiabetic [17], antifungal [18], anti-inflammatory [19], antiprotozoal [20], antituberculosis [21], insecticidal [22], immunomodulatory [23], antiviral, anticancer activities [24], anthelmintic activity [13, 25]. Moreover, there are some reports indicating that some endophytic fungi produce more than twelve metabolites that resemble those produced by the host plants, including alkaloids, flavonoids, saponins, peptides, phenolic acids, terpenes and other active compounds and steroids, and it is considered a promising source for new compounds [26]. Many researchers pointed out that these fungi are chemical factories within the plant and the metabolites of cultured fungi can be alternatives to synthetic compounds and antibiotics, to which the resistance of microorganisms increases over time, as well as their accumulation in the environment without decomposition and their destructive properties on human and/or animal health [27]. To maximize production of bioactive compounds by endophytic fungi, many methods are applied, such as optimization, co-culture, epigenetic, and molecular methods [28].

In this review, factors affecting the biosynthesis of bioactive compounds were discussed, also maximization the production of e bioactive compounds by fungal endophytes was discussed. Furtheromore, this review aims to discuss the biomedical applications of bioactive compounds produced by endophytic fungi.

## Biodiversity of fungal endophytes isolated from plants

Endophytes have been found in all plant sections, including the roots, stems, leaves, fruits, flowers, bark, and scales [29]. Plants represent a reservoir for huge numbers of microorganisms known as endophytes [30]. Endophytes are isolated from plants that growing in different temperate, tropical, semi-tropical, cold, hot and deep sea environments [31]. Also, endophytes can be isolated from marine algae and seaweeds [32]. It is worth noting that more than 300,000 plant species on earth can host endophytes [33], and therefore they constitute a natural source of biological diversity, as many studies of the current century on the biological diversity of endophytes have become more clear as hundreds of genera and species can be isolated from one plant [33]. These large number of isolated endophytic fungi increase the opportunity to obtain new types and strains of endophytic microorganisms that play a major role, regardless of biological diversity. In the extent of the specialization of these organisms, the distribution and density of plant microorganisms are affected by several factors, includes types of agriculture, plant parts, climatic factors, ecological factors and geographic location.

### Types of agriculture

Endophytic fungal community in organic farming significantly higher than conventional agricultural at species diversity level [34, 35]. One reason for this is the alterations in humidity, fertilizer nutrients and fungicides can affect the soil environment in complex [36].

### Plant parts

Endophytic fungi diversity varies through diverse plant tissues, being roots expressively more than shoots or seeds may be due to roots are attached plants with the soil as well as the soil- microbes that could potentially be plant endophyte in herbaceous grassland plants, medicinal plant and rice plants [37]. On the other hand, some fungal endophytes plants can be higher in plant tissues other than roots as zea maize [38].

### Climatic factors

Some endophytic fungi may be affected by environmental conditions such as atmospheric humidity and rainfall, fewer isolates in the winter season compared to other seasons throughout the year [39]. This may be due to some plants may accumulate non-structural carbohydrates under water stress conditions [40].

### Ecological factors

Genetic backgrounds and classification of host plant tissues affect the distribution pattern of fungal endophytes communities [40]

### Geographical location

The difference in geographical location is among the most important factors [41], which also means the different environmental conditions in which plants live leading to the difference in the number and types of internal microorganisms among plants [42, 43].

On the other hand, the diversity and density of microorganisms increase with the age of each member of the plants [44, 45]. Endophytic fungi have been isolated from different medicinal plants. These endophytic fungi produced a wide range of industrially important bioactive compounds. Many entophytic fungi have been isolated from various medicinal plants [46, 47]. These endophytic fungi produced a wide range of biologically active compounds. Aged leaves and petioles were colonized higher by endophytic fungi than by the relatively younger leaves of the endemic plant Cordemoya integrifolia [48].

Most of the isolated endophytic fungi belonging to the phylum Ascomycota and its sexual forms [49]. On the other hand, endophytic fungi can infect a wide range of herbaceous plants spread throughout the world and live a symbiotic life with their hosts [50]. These fungi are known as herbaceous plant fungi [51]. Generally, many genera of *Aspergillus* were isolated from many plants such as *Aspergillus* sp.TRL1 from *Tabebuia rosea* [52] and *Aspergillus* sp. ASCLA from *Callistemon subulatus* [53] and *Aspergillus* sp. GZWMJZ-258 from *Garcinia multiflora* [54] and *Aspergillus* sp. 16-5c from Mangrove [55] and *A. candidus* LDJ-5 from *Rhizophora apiculata*. The fungi isolated from temperate zones often belong to the common fungal genera *Penicillium*, *Alternaria* and *Fusarium* [56–60]. Endophytic species are very diverse. Only a few of the extant inner cells have been described [8, 61].

## Fungal endophytes as a treasure for bioactive compounds

The search for new drugs/pharmaceutical products from microbial origin have been started since the discovery of anticancer drug “Taxol” from Taxomyces andreanae in early 1990’s and Penicillin from *Penicillium notatum* by W. Flemming in 1928 [62]. Both these drugs were isolated from fungi. Initially, taxol was isolated from *Taxus brevifolia* followed by *Taxus wallinchiana*, which harbor endophytic fungi viz. Taxomyces andreanae and Pestalotiopsis microspore, respectively [63]. The discovery of these anticancer drug and antibiotic opened up new vistas to discover new drugs from biological origin. Several antimicrobial compounds produced by endophytic fungi are of importance in their effectiveness against pathogens that have developed resistance to antibiotics. Secondary metabolites from fungal endophytes are strongly affected by many factors, such as the sample collection time, environmental conditions, and site or habitat location of plants (extreme habitats were preferred as saline habitats, very high altitudes, rainforests deserts, swamps and marshes), source of nutrition, tissues of host plant (root, foliar, seeds), types of plant (angiosperms and gymnosperms) [64, 65]. For more, A good physical state of a plant sample with no signs of plant disease is more suitable for selection, soil pH, temperature, humidity, light intensity, soil type, soil microbiota [66].

Several active substances that have antimicrobial effect have been isolated such as linoleic acid, R-glycerol monolinoleate, bisdethio-(bis-methyl-thio)-gliotoxin, fumiquinazoline-F, fumiquinazoline-D, deoxy-thymidine, cerebroside A, (Z,Z).)-N,N0 -[1-[(4-Hydroxy-phenyl)-methylene]-2-[(4-methoxy-phenyl)-methylene]-1,2- ethanediyl]-bisformamide, pyrazoline-3-one trimer, Tricho-9-ene2a,3a,11a,16-tetraol from endophytic fungi* A. fumigatus*. Endophytic fungi have been revealed a powerful reservoir of active natural bio compounds as hexadecanoic acid 2-hydroxy-1ethyl ester, hexadecanoic acid methyl ester, bisabolol oxide B, 9,12-octadecadienoic acid, octadecenoic acid, octadecadienoic acid 2-hydroxy-1ethyl ester, linoelaidic acid, glycidyl palmitate, 9,17-octadecadienal, ethyl-9,12-octadecadienoate, glycidyl oleate; and linoleoyl chloride [67]. Crude ethyl acetate extracts of endophytic Aspergilli have shown promising antibacterial, antifungal activity, beside to revealed antioxidant activity by producing. active secondary metabolites as alkaloids, terpenoids, ρ-terphenyls [67]. These metabolites can be produced in plants either by endogenous cells only or have been transferred to or from the genome of the host plant [68]. One well-known example of the discovery of chemicals derived from endophytic fungi is Taxomyces and reanae isolated from the Pacific yew plant *Taxus brevifolia*. *T. andreaanae* produces paclitaxel, also known as Taxol [69]. This medicine is important for treating cancer [70]. Other indoor plants have since been discovered that also produce paclitaxel in other host species, but to date no successful synthetic source of paclitaxel has been established [8].

Recent studies have revealed the ability of endophytic fungi *Alternaria sp* to produce many active substances that work as Cytotoxic, anti-trypanosomiasis and anti-leishmaniasis Active substances with antifungal activity from endophytic fungi were isolated from *Berkleasmium sp* as Diepoxin, Palmarumycin C11, Palmarumycin C12, Cladospirone B, Palmarumycin C6, 1,4,7β-trihydroxy-8-(spirodioxy-10,80-naphthyl)-7, 8-dihydronaphthalene and Palmarumycin [71]. Endophytes have been shown to create a variety of bioactive compounds (Table 1) that applied in medical science, food, and other fields industries of cosmetics, agriculture. Based on the functional groups the secondary metabolites of this endophytes divided into alkaloids, terpenoids, steroids, polyketones, peptides, flavonoids, furandiones, quinols, perylene derivatives, and depsipeptides xanthones [67, 72].

**Table 1:** Various fungal endophytes and their bioactive compounds

Endophytes	Host plant	Bioactive compounds	References
*A. alternata AE1*	*Azadirachta indica* A. Juss	Phenolics and flavonoids	[73]
A. *alternata KT380662*	*Passiflora incarnata L*	Flavone chrysin (5,7-dihydroxy flavone)	[74]
*Ampelomyces sp*	Urospermum picroides	3-O methylalaternin, altersolanol A, 6-O-methylalaternin andAltersolanol A	[75, 76]
*Aspergillus aculeatus*	*Rosa damascena*	C_32_H_30_O_15_	[77]
A. *awamori*	*Acacia nilotica*	Peptide lectin (N acetylgalactosamine	[78]
A. *flavus*	*Solanum nigrum*	Solamargine	[79]
*A. fumigatus*	sweat potato ( *Ipomoea batatas*)	linoleic acid, R-glycerol monolinoleate, bisdethio-(bis-methyl-thio)-gliotoxin, fumiquinazoline-F, fumiquinazoline-D, deoxy-thymidine, cerebroside A, (Z,Z)-N,N0 -[1-[(4-Hydroxy-phenyl)- methylene]-2-[(4-methoxy-phenyl)-methylene]-1,2- ethanediyl]-bisformamide, pyrazoline-3-one trimer, Tricho-9-ene2a,3a,11a,16-tetraol	[80]
*A. terreus*	*Solanum xanthocarpum* and *Carthamus lanatus* L. (Asteraceae),	Lovastatin, (22E,24R)-stigmasta5,7,22-trien-3-β-, Aspernolides F	[81, 82]
*Berkleasmium sp*	*Dioscorea zingiberensis*	Diepoxin, Palmarumycin C11, Palmarumycin C12, Cladospirone B, Palmarumycin C6, 1,4,7β-trihydroxy-8-(spirodioxy-10,80—naphthyl)-7,8-dihydronaphthalene and Palmarumycin	[71]
*Biscogniauxia mediterranea EPU38CA*	*Echinacea purpurea* (Asteraceae)	5-methylmellein, (3R)-8-hydroxy-6-methoxy-3,5- dimethyl-3,4-dihydroisocoumarin,	[83]
*Botryosphaeria sp*	*Melia azedarach* L and *Huperzia serrata*	Stemphyperylenol, Pycnophorin, Chaetoglobosin C, Djalonensone, Alternariol, β-sitosterol glucoside, 5-hydroxymethylfurfural, Botryosphaerin H. and 13,14,15,16-tetranorlabd-7-en19,6β:12,17-diolide	[84, 85]
*Chaetomium sp*	*Salvia officinalis*	Mollicellin O,Mollicellin H, Mollicellin I Altenuene, 4 -epialtenuene, Aureonitolic acid, Cochliodinol, Isocochliodinol, Iindole-3-carboxylic acid, Cyclo(alanyltryptophane), Orsellinic acid	[86]
*Chaetomium globosum*	*Ginkgo biloba*	Chaetoglobosin and Chaetoglobosin	[87]
*Chaetomium cupreum*	*Macleaya cordata*	Ergosta-5,7,22-trien-3beta-ol	[88]
*Chalara sp*	*Artemisia vulgaris*	Isofusidienol, Isofusidienol, Isofusidienol and Isofusidienol D	[89]
*Cladosporium cladosporioides*	*Huperzia serrata*	Huperzine A, 3-phenylpropionic acid, 5-hydroxyasperentin	[90, 91]
*Coniothyrium sp*	*Salsola oppostifolia*	Coniothyrinones A,B, C and D	[92]
*Colletotrichum gloeosporioides*	*Artemisia mongolica*	Colletotric acid	[93]
*Cryptosporiopsis sp*	*Cryptosporiopsis quercina*	Cryptocandin	[75]
*Cryptosporiopsis quercina*	*Tvipterigeum wilfordii*	Cryptocandin and Cryptocin	[94]
*Curvularia sp*	Asteraceae family	Stemphyperylenol, Murranoic acid, Murranofuran A, Murranopyrone	[95, 96]
*Diaporthe sp*	*Picea mariana* and *Picea rubens needles*	Phomopsolide A,B,C, ascorbic acid, genipinic acid, 4-deoxybostrycin bionectriamide B and trisdechloronornidulin	[97, 98]
*Diaporthe phaseolorum*	*Combretum lanceolatum*	Des-hydroxy Cytochalasin	[99]
*Diaporthe melonis*	*Annona squamosa*	Diaporthemins A, Diaporthemins B, Flavomannin-6,6 -di-O-methyl ether	[100]
*Diaporthe arengae*	*Terminalia arjuna (Roxb.)*	benzene propionic acid, 3, 5-bis (1, 1 dimethylethyl)-4-hydroxy methyl ester and Pterin-6-carboxylic acid and 2, 6-ditert-butyl-4- phenol [ semisolid phenolic compounds]	[101]
*Epicoccum sp*	*Theobroma cacao*	Epicolactone,Epicoccolide A and B	[102]
*Fusarium chlamydosporium*	*Anvillea garcinii*	Fusarithioamide A	[103]
*Fusarium fujikuroi*	*Eleusine coracana*	5-hydroxy 2(3H)-benzofuranone, Harpagoside	[104]
*Fusarium oxysporum*	*Ginkgo biloba*	Cyclosporine, Vincristine	[105]
*Hyalodendriella sp*	*Populus deltoides* Marsh	Hyalodendriol C	[106]
*Lachnum palmae*		Isocoumarin derivatives	[107]
*Lopherdermium nitens*		sesquiterpenoids, Pyrenophorin	[108]
*Nectria sp*	*Acacia nilotica*	phenol-2,6-bis[1,1 dimethylethyl]-4-methyl Citreoisocoumarinol, citreoisocoumarin and macrocarpon C	[78]
*Mucor fragilis*	*Sinopodophyllum hexandrum*	Chaetominine	[109]
*Muscodor albus*	*Cinnamomum zeylanicum*	naphthalene and naphthalene, 1,1-oxybis	[110]
*Mycosphaerella nawae*	*Smilax china*	Amide derivative	[111]
*Nodulisporium sp*	*Erica arborea*	Nodulisporins D, Nodulisporins E, Nodulisporins F, (3S,4S,5R)-2,4,6-trimethyloct-6-ene3,5-diol, 5-hydroxy-2-hydroxymethyl-4Hchromen-4-one, 3-(2,3-dihydroxyphenoxy)- butanoic acid	[112]
*Nodulisporium sp.*	*Ginkgo biloba*	Sporothriolide	[113]
*Oidium sp*	*Terminalia catappa*	Esters of propanoic acid, and butanoic	[114]
*Phaeoacremonium sp*	*Senna spectabilis*	Isoaigialone B, C and Aigialone,	[115]
*Penicillium sp*	*Solanum surattense*	3-O methylviridicatin, Viridicatol, 5-hydroxy-8-methoxy-4- phenylisoquinolin-1(2H)-one	[58]
*P. chrysogenum*	*Ginkgo biloba* and *Cistanche deserticola*	Chrysogenamide A, Circumdatin G, Benzamide, 2 0,30 –dihydrosorbicillin (4) and (9Z,12Z)-2,3- dihydroxypropyloctadeca9,12-dieno, Xanthoviridicatins	[105, 116]
*P. citrinum*	*Nerium oleander L (Apocynaceae)*	15-dimethyl-2-epi-fumiquinazoline A, deoxytryptoquialanone, Citrinadin A and Chrysogenamide A	[117]
*P. raciborskii*	*Rhododendron tomentosum Harmaja*	Outovirin C	[118]
*Periconia sp*	*Taxus cuspidata*	Periconicins A, Periconicins B	[119]
*Pestalotiopsis microspora*	*Cryptosporiopsis quercina*	Ambuic acid	[75]
*Pestalotiopsis theae*	*Cistanche deserticola*	Pestalotheol A, B, C and D	[116]
*Pezicula sp*	*Forsythia viridissima*	Mellein	[120]
*Phialophora mustea*	*Crocus sativus*	Phialomustin C, Phialomustin D	[121]
*Plectophomella sp*	*Artimisia maritime*	Mycorrhizin A, Cytochalasins E, Cytochalasins K, Radicinin	[122, 123]
*Phyllosticta spinarum*	*Platycladus orientalis*	Tauranin, ( +)-(5 S,10 S)-40 –hydroxy methylcyclozonarone, 3-ketotauranin, 3alpha-hydroxytauranin, 12-hydroxytauranin, Phyllospinarone	[124]
*Rhizophora mucronata*	*Chinese mangrove*	Pestalotiopsone F	[125]
*Seimatosporium sp*	*Rosa varieties*	Seimatorone	[126, 127]
*Stemphylium globuliferum*	*Mentha pulegium*	Alterporriol G and H (mixture)Altersolanol KAltersolanol LStemphypyrone	[128]
*Syncephalastrum sp*	*Acacia nilotica*	herbarin, Naphthoquinones (O-phenethylherbarin), and herbaridine	[78]
*Talaromyces purpureogenus*	seaweed Grateloupia filicina C. Ag (Wulf.)	Talaromyolide K	[129]
*Tibouchina granulosa*	*Combretum lanceolatum*	Brefeldin and heptelidic acid	[99]
*Trichoderma sp*	*mangrove*	Dichlorodiaportin, Dichlorodiaportinolide pregnane-3,20β-diol, 14α,18α-[4- methyl-3-oxo-(1-oxa-4-azabutane-1,4-diyl)], diacetate; 4-piperidineacetic acid,1- acetyl-5-ethyl-2-[3-(2- hydroxyethyl)-1-H-indol-2-yl]-a- methyl, methyl ester; corynan-17-ol, 18,19-didehydro-10-methoxy and oleic acids	[130, 131]
*Trichoderma brevicompactum*	*Garlic butter*	Trichodermin	[132]
*Trichoderma koningiopsis*	*Artemisia argyi*	Koningiopisin C	[130, 133]
*Trichothecium sp*	*Phyllanthus amarus*	Trichothecinol A	[134]
*Xylaria sp*	*Alibertia macrophylla*	Griseofulvin, Cytochalasin	[135–137]

## Enhancement of secondary metabolites biosynthesis in fungal endophytes

**Fig. 1:**
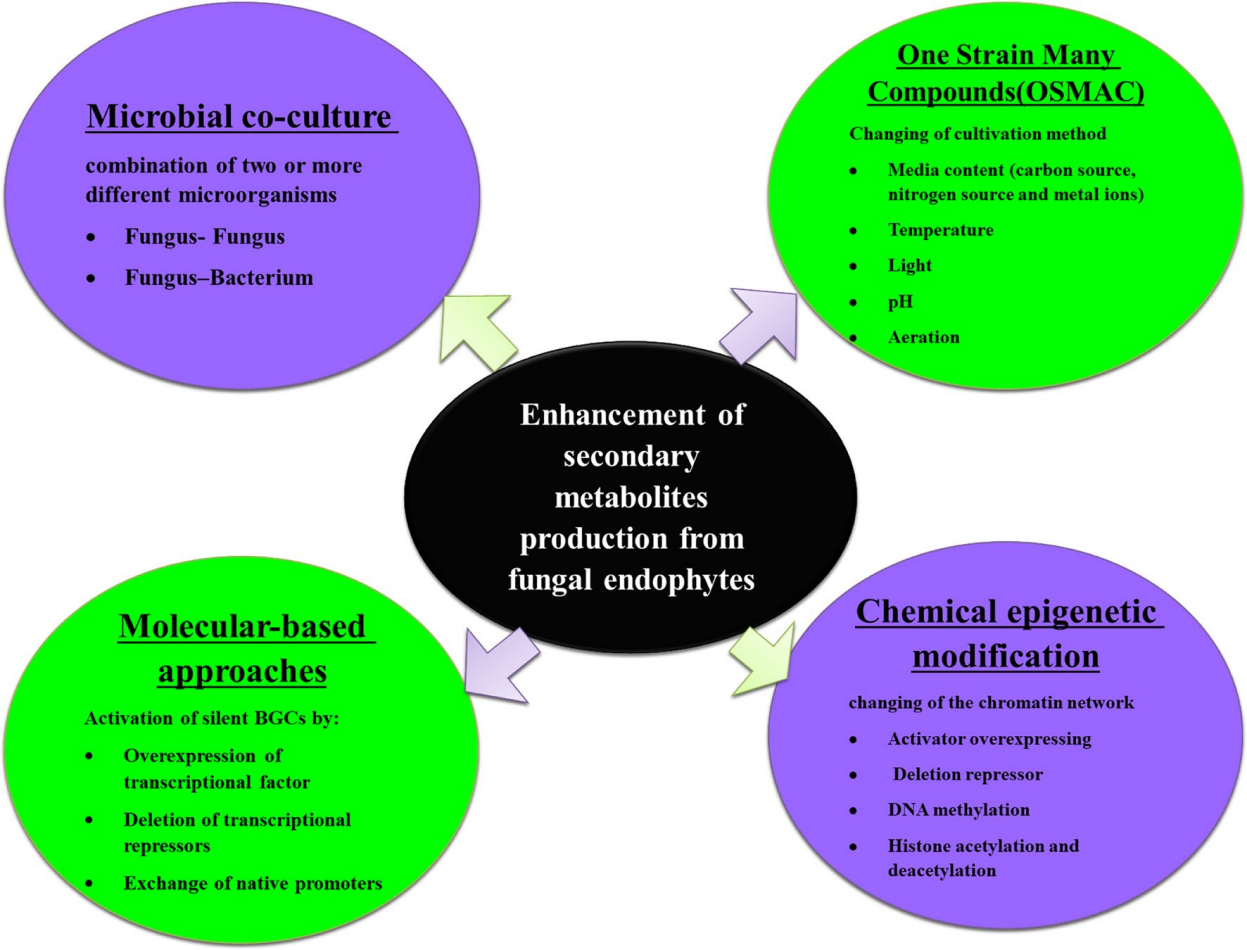
Enhancement of Secondary Metabolites Biosynthesis in Fungal Endophytes

There are many methods for enhancing biosynthesis of secondary metabolites in endophytic fungi such as optimization, co-culture, epigenetic modification and molecular methods as shown in Fig. 1.

### Optimization method

One Strain, Many Compounds (OSMAC) is an important method to enhance secondary metabolite from fungal endophytes or produce different compounds by changing of cultivation method such as temperature, light, pH, agitation and media that lead to changing secondary metabolite [28]. OSMAC approach use changing of cultivation method such as changing of media content (carbon source, nitrogen source and metal ions), physical properties (temperature, pH and aeration) or addition some molecules (enzyme activation / inhibition, MgSO_4_, NaCl) to induce or produce a new secondary metabolite [138]. Extraction of different secondary metabolites (four chaetomugilins, two malforms and seven chaetoglobosins) from fungal endophyte (*Chaetomium* sp.) which isolated from *Astragalus membranaceus* by OSMAC strategy after changing growth media for fungal cultivation [139]. Supratman et al. [140] isolated endophytic *Clonostachys rosea* from mangrove plants and applied OSMAC strategy where alters of rice media by adding of apple juice to produce different secondary metabolites such as vertinolide, dihydrovertinolide and clonostach acids. Potato dextrose agar (PDA) media was developed through OSMAC to produce a new secondary metabolite (eight indole diketopiperazines, five quinazolinone alkaloids and three helvolic acid) by endophytic *A. fumigatus* which isolated from *Astragalus membranaceus* [141]. Moreover, solid rice medium which includes inducer compounds (NaNO_3_ or monosodium glutamate) led to produce eleven new lactam derivatives, pramanicin A and aplosporellins A-K from endophytic *Aplosporella javeedii* through OSMAC method [142]. Furthermore, kojic acid was produced from endophytic *A. flavus which* isolated from leaves of *Annona squamosal* by OSMAC strategy on different cultivation media (rice media, sweet and waxy corn media), this can be used in many applications in medical, cosmetic and industry [143].

### Co-culture method

The combination of two or more microorganisms in the same media to enhance production of secondary metabolite or activate of cryptic genes called Co- culture strategy [144]. In this method, an artifcial microbial community is constructed to activate the biosynthetic gene clusters to produce new compounds. Co-culture method is classified to fungus–bacterium, fungus–fungus and bacterium–bacterium that make enhancement or production of new compound and activation of silent gene clusters for microorganism [145].

#### Fungus–fungus

In this technique, co-culturing is carried out by combination of two fungal species to enhance or produce new products [146]. In a previous study, co-culture was carried out between endophytic *Phoma sp*. with *Armillaria sp.* to produce new five secondary metabolites product, two phenolic compounds (phexandiols), three aliphatic ester derivatives (phomesters) [147]. Li et al. [148] isolated eight new compounds from co-culturing of *Armillaria sp. and Epicoccum sp.*, and only one compound has anticancer activity.Murakami et al. [149] induced production of new secondary metabolites through co-culture between *Talaromyces pinophilus* and *Paraphaeosphaeria sp.* Co-culture between two endophytic fungi *Fusarium tricinctum* and *F. begonia* led to produce novel compounds subenniatin A and B [150].

#### Fungus–bacterium

Co-culturing in this case is between different microorganism (fungi and bacteria) in the same medium. Sun et al. [151] carried out co-culture between *Aspergillus sydowii* and* Bacillus subtilis*, and found seven new compounds which possess biological activities. Akone et al. [152] reported that, co-culturing of endophytic *Chaetomium sp.* with* B. subtilis* on rice medium led to increase in the produced metabolites (8.3 fold), some of these metabolites have anticancer and antibacterial activities. Moreover, co-culturing was carried out between *Bionectria* sp either with *Bacillus subtilis* or with *Streptomyces lividans, where* two new *o*-aminobenzoic acid derivatives *were produced* [153].

### Chemical epigenetic modification

Gene silencing or activation through control of chromatin level is considered one of the mechanisms that regulate biosynthesis of fungal secondary metabolites [154]. Epigenetic modification method uses small molecules that change the chromatin network and alter of secondary metabolite profile to enhancement production of secondary metabolite or induction of silent biosynthetic gene cluster [155]. Epigenetic modification method can act activator overexpressing or deletion repressor to some type, cause genetic change that enhancement production of secondary metabolite [156]. Histones have an important role of post translation modification that include methylation, phosphorylation, methylation, citrullination, ADP-ribosylation and ubiquitination that make changing via interfare with DNA and nuclear proteins [157]. One or more of the chemical epigenetic modifiers such as DNA methyltransferases (DNMT) inhibitors and histone deacetylases (HDAC) inhibitors and were added to medium through cultivation of fungi to achieve chemical epigenetic modification [158]. Addition of these compounds to growth culture leads to activate or suppress some enzymes which have ability to induce production of new compounds. Histone deacetylases inhibition was performed through epigenetic modification, where both suberoylanilide hydroxamic acid and nicotinamide were added to growth medium of *Penicillium brasilianum* [159]. Sharma et al. [160] used valproic acid as chemical modifier to growth culture of *Diaporthe* sp., where this induced production of new compounds as xylarolide A, xylarolide Band diportharine A through inhibition of Histone deacetylases. Likewise, histone deacetylase inhibition was carried out through addition of valproic acid, this led to increase production of fumiquinazoline C to tenfold in *Aspergillus fumigatus* [161]. Wu et al. [162] succeeded in chemical epigenetic modification of *Cochliobolus lunatus,* where added 5-azacytidine to produce new metabolites (α-pyrones, cochliobopyrones, isocoumarins and chromone).Trichostatin A as histone deacetylase inhibitor was added to endophytic *Bjerkandera adusta* HS-28, where induced production of tremulane sesqiterpenoids [163]. Li et al. [164] applied chemical epigenetic modification on endophytic *Eupenicillium sp.* through adding nicotinamide as histone deacetylase inhibitor which induced production of eupenicinicol C and D as novel compounds. The mechanism behind the activation of silent biosynthetic pathways during co-cultivation can be explained by either unilateral stimulation through physical interaction or chemical signals; or by unilateral induction of the biosynthesis of signaling molecules which then trigger the production of cryptic metabolites [165].

### Molecular-based approaches

This method is used to activate silent biosynthetic gene clusters (BGCs) and enhancement production of secondary metabolite via using different pathway such as genetic engineering and mining of target strain for gene cluster of secondary metabolite by using bioinformatics [166]. Pathway-specific regulatory genes where located in or outside for specific BGC are include particular BGC with the inactivation or repression of biosynthesis of secondary metabolites also stimulate transcription factor that regulates genes of secondary metabolites [167]. Different strategies are used to activate cryptic BGC and enhancement production of secondary metabolites in fungi such as overexpression of transcriptional factor, deletion of transcriptional repressors and the exchange of native promoters with inducible or constitutive promoters [168]. This approach allows an understanding of the target substrate and physicochemical properties of the end products [169]. Bergmann et al. [170] applied strategy for induction of silent pathways in *A. nidulans* to produce novel two PKS-NRPS hybrid metabolites (aspyridone A and B) through overexpression of transcription factor gene Apda.

## Biomedical applications of fungal endophytes

### Antimicrobial activity

The emergence of pathogenic bacteria and fungi resistant to commercial drugs is a relevant problem faced by health services, this due to the microbes acquiring new mechanisms to resist antimicrobial agents [171, 172]. Therefore, the discovery of effective antimicrobial agents is required. Fungal endophytes can live in plant tissues without producing any apparent symptoms or obvious harmful effects to their hosts [67]. Fungal endophytes are considered one of important reservoirs of bioactive compounds which have different biological activities such as antimicrobial, antioxidant, anticancer, antiviral activities. There are many active secondary metabolites including steroids, flavonoids, terpenoids, peptides, quinones, lignans, alkaloids, phenylpropanoids, phenolics, isocoumarins which produced from endophytic fungi have a great activity against diverse pathogenic microorganisms [173]. Therefore, the extraction of new antimicrobials from new fungal endophytes is reqiured to combat antimicrobial resistance [174]. Aspergillus is the most genus among other fungal endophytes isolated in the period 2019 to 2022 according to data shown in Table 2. *Aspergillus sp. ASCLA* was isolated from leaf tissues of the medicinal plant *Callistemon subulatus* and Isoshamixanthone was isolated which have antimicrobial activity against pathogenic micororganisms [53]. Moreover, Sharaf et al. [67] isolated *A. flavus* from *Ocimum Basilicum* and found it has antibacterial and antifungal activities against *Staphylococcus aureus, Bacillus cereus, B. subtilis, Escherichia coli, Salmonella typhimurium, Pseudomonas aeruginosa, Klebsiella pneumonia,* and* Candida albicans* at a concentration of 1000 µg/mL. Furthermore, *A. fumigatus* was isolated from leaves of *Albizia lucidior* [175]* and Ocimum Basilicum* [67], where reported that *A. fumigatus* has potential antibacterial and antifungal activities against most common resistant microbes. The fungal extract of endophytic *A. niger* which isolated from *Sonneratia apetala* exhibited antimicrobial activities against *E. coli NCTC 12241, S. aureus NCTC 12981, M. lutus NCTC 7508, P. aeruginosa NCTC 7508 and C. albicans ATCC 90028 *[176]. Also, *A. nidulans* was isolated from *Ocimum Basilicum* which has antimicrobial against many resistant microbes [67]. Elkhouly et al. [177] separated anofinic acid from endophytic *A. tubenginsis* which has promising antimicrobial activity gram positive, gram negative and unicellular fungi, also could inhibits biofilm formation. Also, Mohamed et al. [178] separated aspergillethers A and B as a new diaryl ether derivatives from A. versicolor isolated from the roots of *Pulicaria crispa* Forssk (Asteraceae) which has strong antimicrobial activity against *S. aureus*, *B. cereus*, and *E. coli*,* C. albicans* and *Geotrichium candidum*. Maliehe et al. [179] isolated novel endophytic *A. welwitschiae* from *Aloe ferox* Mil, also reported that *A. welwitschiae* has potential antibacterial activity against pathogenic microbes.

**Table 2:** Biological activities of endophytic fungi isolated in the period 2019–2022

Genera	Endophytic fungi	Host plant	Biological activity	Refs.
*Aspergillus*	*Aspergillus sp.TRL1*	*Tabebuia rosea*	Anticancer activity	[52]
	*Aspergillus sp. ASCLA*	*Callistemon subulatus*	Antimicrobial and anticancer activities	[53]
	*Aspergillus sp. GZWMJZ-258*	*Garcinia multiflora*	Anticancer activity	[54]
	*Aspergillus spp.*	*Anthopleura* *xanthogrammica*	Antimicrobial activity	[211]
	*Aspergillus sp. 16-5c*	*Mangrove*	Antidiabetic activity	[55]
	*A.candidus LDJ-5*	*Rhizophora* *apiculata Blume*	Anticancer activity	[212]
	*A.flavus*	*Tylophora ovata*	Anticancer activity	[213]
	*A. flavus*	*Ocimum Basilicum*	Antimicrobial and antioxidant activities	[67]
	*A.flavus*	*Ficus elastic*	Anticancer activity	[214]
	*A.flocculus*	*Markhamia platycalyx*	Anticancer and anti-trypanosome	[215]
	*A.fumigatus*	*Albizia lucidior*	Antibacterial activity	[175]
	*A. fumigatus*	*Ocimum Basilicum*	Antimicrobial and antioxidant activities	[67]
	*A.micronesiensis*	*Kappaphycus alvarezii*	Anticancer activity	[216]
	*A. minisclerotigens*	*Mangifera casturi* *Kosterm*	Antioxidant activity	[217]
	*A.neoniger*	*Ficus carica*	Antimicrobial and anticancer activities	[218]
	*A.nidulans*	*Passiflora Incarnate*	antioxidant activity	[219]
	*A.nidulans*	*Ocimum Basilicum*	Antimicrobial and antioxidant activities	[67]
	*A.niger*	*Cocos nucifera (L.)*	Antimicrobial activity	[207]
	*A. niger*	*Sonneratia apetala*	Antimicrobial activity	[176]
	*A. oryzae. (R2MC3A)*	*Aquilaria.microcarpa*	Antifungal and antioxidant activities	[220]
	*A. oryzae*	*Mangifera casturi* *Kosterm*	Antioxidant activity	[217]
	*A.terreus*	*Ficus elastica*	Anticancer activity	[214]
	*A. terreus*	*Artemisia annua*	Antioxidant activity	[221]
	*A. Tubenginses ASH4*	*Hyoscyamus muticu*	Antimicrobial, antibiofilm, antioxidant and Anticancer Activity	[177]
	*A. versicolor*	*Pulicaria crispa Forssk*	Antimicrobial activity	[178]
	*A.welwitschiae*	*Aloe ferox Mill*	Antibacterial activity	[179]
*Penicillium*	*Penicillium sp.*	*Stephania Dielsiana*	Antimicrobial activity	[56]
	*Penicillium vsp. ct-28*	*Corydlis tomentella*	Anticancer activity	[57]
	*P. roqueforti*	*Solanum surattense*	Antioxidant activity	[58]
	*P. citrinum*	*Azadirachta indica*	Antimicrobial activity	[180]
	*P. ochrochloronthe*	*Taxus media*	Antimicrobial and anticancer activities	[181]
	*P. funiculosum*	*Ficus elastic*	Antimicrobial activity	[182]
	*P. simplicissimum*	*Loranthus micranthus*	Antimitotic, anti-inflammatory and anticancer activities	[222]
	*P. pinophilum*	*Alloteropsis cimicina*	Antimicrobial and antioxidant activities	[183]
*Alternaria*	*Alternaria sp. MG1*	*Vitis quinquangularis*	Anticancer activity	[59]
	*Alternaria sp. SZMC 23772*	*Hypericum perforatum*	Antimicrobial activity	[60]
	*Alternaria sp. sb23*	*Schisandra sphenanthera*	Anticancer activity	[223]
	*Alternaria sp. LV52:*	*Cystoseira tamariscifolia*	Anticancer activity	[224]
	*A. alternata*	*Paeonia lactiflora*	Anticancer activity	[225]
	*A. alternata MGTMMP031*	*Vitex negundo*	Anticancer activity	[226]
	*A. altenata*	*Lithospermum* *Officinale*	Antioxidant activity	[227]
	*A.alternata*	*Picrorhiza kurroa*	Antioxidant and antimicrobial activities	[185]
	*A.alternata*	*Melissa officinalis*	Anticancer activity	[228]
	*A.alternata*	*Ziziphus spina-christi*	Antimicrobial and antioxidant activities	[186]
	*A. brassicicola*	*Terminalia arjuna*	Anticancer activity	[229]
	*A.Destruens*	*Calotropis gigantean*	Antimicrobial, antibiofilm, antidiabetic activities	[230]
	*Alternaria tenuissima PE2*	*Psidium guajava L*	Antimicrobial activity	[190]
	*F. equiseti*	*Sonneratia apetala*	Antimicrobial activity	[176]
	*F. proliferatum*	*Cissus quadrangularis L*	Antimicrobial activity	[187]
	*Fusarium sp.*	*Physalis angulata L*	Antibacterial and antioxidant activities	[188]
	*F. oxysporum*	*Otoba gracilipes*	Antioxidant activity	[231]
	*F. oxysporum*	*Sceletium tortuosum L*	Antibacterial activity	[189]
*Chaetomium*	*Chaetomium sp.*	*Avicennia marina*	Antimicrobial and antioxidant activities	[173]
	*Chaetomium sp. HQ-1*	*Astragalus chinensis*	Antimicrobial activity	[191]
	*Chaetomium sp. SYP-F7950*	*Panax notoginseng*	Antimicrobial and anticancer activities	[192]
	*C. cruentum*	*Conyza blinii H. Lév*	Antioxidant activity	[232]
	*C.globosum*	*Litsea cubeba*	Antimicrobial activity	[193]
	*C.globosum*	*Moringa oleifera*	Antimicrobial and antibiofilm activities	[194]
*Trichoderma*	*T. Harzianum*	*Rosmarinus Officinalis*	Antimicrobial activity	[195]
	*T. Harzianum*	*Kadsura angustifolia*	Antiviral activity	[233]
	*T. harzianum*	*Zingiber officinale*	Anticancer activity	[234]
	*T. harzianum*	*Ficus elastica*	Antimicrobial activity	[182]
	*T. cf. harzianum*	*Chloranthus japonicus*	Antimicrobial activity	[235]
	*T. virens QA-8*	*Artemisia argyi*	Antimicrobial activity	[196]
	*T. koningii CSE*	*Cupressus sempervirens*	Antifungal activity	[197]
	*T. atrovirid*	*Juniperus communis*	Antifungal activity	[197]
*Diaporthe*	*Diaporthe sp.,*	*Cinnamomum Loureiroi*	Antimicrobial and antioxidant activities	[198]
	*D. eres*	*Ligustrum obtusifolium*	Antioxidant and anticancer activities	[236]
	*D. phaseolorum,*	*Stephania Dielsiana*	Antimicrobial activity	[56]
	*D. terebinthifolii*	*Schinus terebinthifolius)*	Antimicrobial, activity	[199]
*Nigrospora*	*N. sphaerica*	*Helianthus annuus*	Antimicrobial and anticancer activities	[200]
	*N. sphaerica*	*Dillenia indica L*	Antimicrobial activity	[201]
	*N. sphaerica*	*Bruguiera gymnorrhyza*	Antimicrobial, anti-cancer, anti-inflammatory and α-glucosidase inhibitory activities	[202]
	*N. sphaerica*	*Adiantum philippense L*	Antimicrobial, activity	[203]
	*N.sphaerica*	*Euphorbia hirta (dudhi) L*	Antioxidant activity	[237]
	*N.oryzae*	*Tinospora cordifolia*	Antioxidant activity	[238]
*Epicoccum*	*E. Nigrum*	*Hypericum Perforatum*	Antimicrobial activity	[60]
	*E. nigrum*	*Salix sp.*	Anticancer activity	[234]
	*E. nigrum*	*Terminalia arjuna*	Anticancer activity	[239]
	*E. nigrum SCNU-F0002*	*Acanthus ilicifolius L*	α-glucosidase inhibitory and antioxidant activity	[240]
*Colletotrichum*	*Colletotrichum sp.*	*Physalis angulata L*	Antibacterial and antioxidant activities	[188]
	*Colletotrichum sp.*	*Zanthoxylum oxyphyllum* *Edgew*	Antimicrobial activity	[241]
	*Colletotrichum sp.*	*Stephania Dielsiana*	Antimicrobial activity	[56]
	*C. gloeosporioides*	*Sonneratia apetala*	Antimicrobial activity	[176]
	*C. coccodes*	*Houttuynia cordata*	Antimicrobial activity	[242]
*Phoma*	*Phoma sp.*	*Aconitum vilmorinianum*	Antiviral activity	[243]
	*P. bellidis*	*Tricyrtis maculata*	Anticancer activity	[244]
	*P. macrostoma*	*Glycyrrhiza glabra Linn*	Anticancer activity	[245]
*Emericella*	*Emericella sp. TJ29*	*Hypericum Perforatum*	Anticancer activity	[246]
	*E. nidulans (E6658*	*Pelargonium graveolens*	Antimicrobial activity	[184]
*Curvularia*	*Curvularia sp.*	*Phyllanthus niruri L*	Antioxidant and anticancer activities	[247]
	*Curvularia lunata*	*Ficus religiosa L*	Antioxidant and antidiabetic activities	[55]
	*Curvularia papendorfii*	*Vernonia amygdalina*	Antibacterial and antiviral activity	[248]
Other genera	*Chaetosphaeronema hispidulum*	*Bassia dasyphylla*	Anticancer activity	[249]
	*Cladosporium tenuissimum*	*Swietenia mahagoni*	Antioxidant activity	[250]
	*Bipolaris sp. L1–2*	*Lycium barbarum*	Anticancer activity	[251]
	*Myrothecium roridum spp.*	*Trachelospermum* *Jasminoides*	Anticancer activity	[252]
	*Neosartorya fischeri JS0553*	*Glehnia littoralis*	Antioxidant activity	[253]
	*Neurospora tetrasperma*	*Cordyline fruticose*	Antimicrobial activity	[204]
	*Leptosphaeria sp. XL026*	*Panax notoginseng*	Antimicrobial activity	[205]
	*Paraphaeosphaeria sp. F03*	*Paepalanthus planifolius*	Antimicrobial and anticancer activities	[206]
	*Raffaelea sp.*	*Cocos nucifera (L.)*	Antimicrobial activity	[207]
	*Hypomontagnella* *Monticulosa*	*Zingiber griffithii*	Anticancer activity	[254]
	*Lasiodiplodia venezuelensis*	*Syzygium samarangense L*	Antioxidant activity	[255]
	*Schizophyllum commune*	*Aloe vera*	Antidiabetic activity	[256]
	*Phomopsis sp.*	*Polygonatum cyrtonema Hua*	Anticancer activity	[257]
	*Pseudopestalotiopsis. camelliae-sinensis*	*Justicia gendarussa*	Antimicrobial, Antioxidant activities	[208]
	*Phyllosticta capitalensis*	*Bruguiera sexangula*	Antimicrobial and anticancer activities	[209]
	*Fomitopsis meliae*	*Dillenia indica L*	Antimicrobial activity	[210]
	*Paraphaeosphaeria sp. F03*	*Paepalanthus planifolius*	Antimicrobial and anticancer activities	[206]
	*Pestalotiopsis sp.*	*Leaves of tea tree*	Anticancer activity	[258]

Penicilli is another group of endophytic ascomycetes which is common live inside plants. Recent studies reported that penicilli have potential antibacterial and antifungal activities toward resistant microbes. Endophytic *Penicillium sp.* was isolated form the host plant *Stephania dielsiana* and tested against seven different pathogenic bacteria and showed promising antimicrobial activity [56]. Kumari et al. [180] reported that *P. citrinum* from* Azadirachta indica* has potential antimicrobial activity toward human pathogenic bacteria and fungi. Moreover, Zhao et al. [181] separated three new derivatives of α-pyrone from *P. ochrochloronthe* isolated from *Taxus media*, these derivatives have antimicrobial activity against some pathogenic bacterial and fungal strains with MIC in range 12.5–100 µg/ml. Furthermore, *P. funiculosum* [182] and *P. pinophilum* [183] were isolated from *Ficus elastic* and *Alloteropsis cimicina* respectively*,* where their extracts exhibited the extract exhibited potential antimicrobial activity toward clinical bacterial strains. Yasser et al. [184] isolated *Emericella nidulans* from* Pelargonium graveolens,* which has strong antifungal activity against* Microsporum audouinii, A. niger and Penicillium sp.*

*Alternaria spp*. have promising antimicrobial activity as shown in Table 2. *Alternaria* sp. SZMC 23772 was isolated from medicinal herb *Hypericum perforatum,* where new metabolite emodin which has antimicrobial activity against some human pathogenic fungi was separate*d* [60]. Chandra et al. [185] reported that, the extract from *A. alternata* which isolated from *Picrorhiza kurroa* has antibacterial activity against *B. subtilis* and *S. aureus.* Elghaffar et al. [186] reported that, ethyle acetate crude extract of *A. alternata* exhibited promising antimicrobial activity against Gram-negative bacteria (*E. coli* ATCC 11229, *Proteus vulgaris* RCMB 004, *P. aeruginosa* ATCC 27853, and *Klebsiella pneumonia* RCMB 003), Gram-positive bacteria (*B. subtilis* RCMB 015, *S. aureus* ATCC 25923, and *S. epidermidis* ATCC 14990), and unicellular fungi (*Candida albicans* ATCC 90028), this activity may be attributed to presence alkaloids, tannins, flavonoids, glycosides, phenols, and terpenoids in the crude extract of *A. alternata.* Moreover, the extract of endophytic *A. Destruens* isolated from *Calotropis gigantean* has ability to inhibit many pathogenic microbial strains. As well, there are many *Fusarium spp.* have antimicrobial activity against bacterial and fungal strains. The crude extract of endophytic *F. equiseti* isolated from* Sonneratia apetala* showed antimicrobial activity against most common pathogenic bacteria and fungal strains [176]. Singh et al. [187] isolated *F. proliferatum* from medicinal plant *Cissus quadrangularis* L., and found the extract exhibited antibacterial activity against pathogenic bacteria where MIC was 40–120 µg/ml. Furthermore, *Fusarium sp.* was isolated from stem of *Physalis angulata L.,* where the extract has antibacterial activity toward *E. coli* and *S. aureus* with minimum inhibitory concentration (MIC) value ranging from 8 to 64 μg/mL [188]. Moreover, Manganyi et al. [189] isolated endophytic *F. oxysporum from Sceletium tortuosum L.,* and proved the extract has antibacterial activity against *Enterococcus faecalis* and *E. gallinarum*while *B. cereus. *Chatterjee et al. [190] isolated endophytic *A. tenuissima* PE2 from common fruit plant *Psidium guajava* L., EA extract of the cell free supernatant of *A. tenuissima*was found effective against both Gram-positive and Gram- negative bacteria with MIC values of ∼500 µg/mL and ∼800 µg/mL, respectively.

*Chetomium and trichoderma* genera are considered the common fungal endophytes among other fungi. Endophytic *chetomium* spp. were isolated from different plant species as *Avicennia marina* [173], *Astragalus chinensis* [191] and *Panax notoginseng* [192], where have antimicrobial activity toward human pathogenic bacterial and fungal strains. Moreover, endophytic *C. globosum* which was isolated from *Litsea cubeba* [193] and *Moringa oleifera* [194] have outstanding antimicrobial and antibiofilm activities.

*Trichoderma harzianum was isolated from Rosmarinus Officinalis* where exhibited significant antimicrobial activity against *P. aeruginosa, S. aureus, K. pneumonia, B. subtilis* and* E. coli* [195]. Likewise, *T. harzianum* was isolated from Ficus elastic and appeared antibacterial activity where two new isocoumarin derivatives (1 and 2) were separated from *T. harzianum* where exhibited antibacterial activity against *E. coli* [182]. Furthermore, Trichocadinins B-G and new cadinane-type sesquiterpene derivatives were separated from *T. virens *which isolated from* Artemisia argyi,* these compounds have antibacterial and antifungal activities [196]. Erfandoust et al. [197] isolated *T. koningii and T. atrovirid* from*Cupressus sempervirens* and* Juniperus communis* respectively, where both exhibited strong antifungal activity against human pathogenic* A. fumigatus and A. flavus.*

*Endophytic Diaporthe spp.* have different biological activities particularly antimicrobial according to Table 2. *Diaporthe sp.* was isolated from* Cinnamomum Loureiroi* where has antibacterial activity against *B. cereus* and *S. epidermidis* with MIC 3.91 μg/mL [198]. Moreover, *D. phaseolorum*and* D. terebinthifolii* were isolated from *Stephania Dielsiana* [56] and* Schinus terebinthifolius* [199], where both species have antimicrobial activity against human pathogenic bacterial and fungal strains. Likewise, *Nigrospora spp. have antimicrobial activity against pathogenic microbes,* Supaphon, Preedanon [200] isolated *N. sphaerica* from *Helianthus annuus* which has antibacterial activity against *S. aureus* and methicillin-resistant *S. aureus* (Gram-positive bacteria) with MIC in the range of 16–32 μg/mL. Moreover, *N. sphaerica was isolated from Dillenia indica L.* [201],* Bruguiera gymnorrhyza* [202] and *Adiantum philippense L.* [203], the three studied reported that all extracts showed strong antibacterial and antifungal activities toward common pathogenic bacterial and fungal strains.

*Colletotrichum spp.* have ability to produce bioactive compounds which could inhibit pathogenic microbes. *Colletotrichum spp.* were isolated from *Physalis angulata L* [188] and *Stephania Dielsiana* [56], which have promising antimicrobial against human pathogenic bacterial and fungal strains with low MIC. As well, Among *Epicoccum spp., E. nigrum* is the most common as fungal endophyte which have novel bioactive compounds*.* Vigneshwari et al. [60] reported that, *E. nigrum which isolated from Hypericum perforatum* could produce both emodin and hypericin which has potential antimicrobial activity against most common pathogenic microbes.

Other endophytic fungi as *Neurospora tetrasperma *[204]*, Leptosphaeria sp. XL026 *[205]*,* *Paraphaeosphaeria sp. F03* [206]*, Raffaelea sp.* [207]*, Pseudopestalotiopsis camelliae-sinensis* [208]*, Phyllostictacapitalensis* [209]*,* and *Fomitopsis meliae* [210] has been reportedas antimicrobial agents against human pathogenic bacterial and fungal strains.

### Antiviral activity

Viruses cause serious outbreaks in all continents leading to difficult symptoms and mortality, and enormous economic burden for society. In addition, the constant emergence of new serotypes in virus groups that have a high mutation rate and low fidelity for viral replication adds challenges in combatting against these viruses. New viruses emerge all the time and presently we have limited number of vaccines and only few antivirals to combat viral diseases. There is a global need for new antiviral compounds to solve drug resistance problems. The resistance of human disease to well-known (commercial) antibiotics is increasing rapidly nowadays, so discovering new alternative agents is indispensable required for management those maladies. Bioactive compounds isolated from natural biological sources offer a vast and unexplored diversity of chemical structures, unmatched by even the biggest combinatorial databases [33]. Recently, it was reported that an endophytic *T. Harzianum* was isolated from* Kadsura angustifolia,* where nigranoic acid was separated, where reported that nigranoic acid has strong antiviral activity where inhibits HIV-1 reverse transcriptase [233]. Khiralla et al. [248] isolated *Curvularia papendorfii* from* Vernonia amygdalina,* and found the crude extract of C. papendorfii showed antiviral effect against coronavirus with reduction 40% of the virus-induced cytopathogenic effect at lower multiplicity of infection. *Phoma sp.* was isolated from *Aconitum vilmorinianum, and new* rare 14-nordrimane sesquiterpenoid (phomanolide) was separated [243]. Phomanolide exhibited strong antiviral activity against influenza A virus (A/Puerto Rico/8/34, H1N1) with IC_50_ values of 2.96 ± 0.64 and 20.98 ± 2.66 μg/mL, respectively.

### Antioxidant activity

The significance of antioxidant-active substances originates from their capacity to protect cells from damage produced by reactive oxygen species (ROS) and oxygen-derived free radicals, which contribute to a wide range of adverse effects such as DNA damage, carcinogenesis, and cellular degeneration [259, 260]. ROS causes many diseases such as cancer, cardiovascular disease, ischemia, Alzheimer, diabetes mellitus, hypertension, and ageing [261]. Antioxidants can be present in a variety of medicinal plants, vegetables, and fruits. Furthermore, metabolites of fungal endophytes have been found to represent a possible source of new natural antioxidants. Recent studies confirmed that Aspergilli are the most common fungal endophytes for antioxidant production as shown in Table 2. Sharaf et al. [67] reported that, *A. flavus, A. fumigatus* and* A. nidulans* have promising antioxidant activity where IC50 was in range 68.4–347.1 µg/ml. Nuraini et al. [217] isolated *Aspergillus minisclerotigens* AKF1 and *Aspergillus oryzae* DK7 from *Mangifera casturi Kosterm* and found the both fungi exhibited antioxidant activity with IC50 142.96 and 145.01 µg/mL, respectively. da Silva et al. [219] reported that, the extract of endophytic A. nidulans isolated from *Passiflora Incarnate* has potential antioxidant activity. Moreover, *A. oryzae* and *A. terreus* have antioxidant activity [221]. Likewise, antioxidant activity of endophytic penicillium spp. has been reported. Ikram et al. [58] confirmed that, the extract of P. *roqueforti contains* ferulic acid, cinnamic acid, quercetin, and rutin which are promising as antioxidants. Nischitha, Shivanna [183] illustrated endophytic *P. pinophilum* is a natural reservoir of novel bioactive compounds with antimicrobial and antioxidant properties. Moreover, A. alternata among endophytic Alternaria spp. is the common for antioxidant compounds production [186]. Furthermore, endophytic fusarium spp. as *F. tricinctum, F. oxysporum* [231]* and Fusarium sp.* [188] were reported as antioxidant agents. Endophytic *Chaetomium* is considered one of the most fungal endophytes which produce antioxidant compounds [262]. Zhao et al. [232] separated flavonoids from *C. cruentum* which isolated from *Conyza blinii H. Lév,* where exhibited promising antioxidant activity. Furthermore, endophytic *Diaporthe* have been reported for antioxidant activity [263]. *Epicoccum* and *Nigrospora* genera have potential antioxidant activity as *E. nigrum *[240], *N. sphaerica* [237] and *N. oryzae *[238]. Moreover, endophytic *Colletotrichum sp. *[188], *Curvularia sp.*[247] and *Curvularia lunata* [55] have been reported as antioxidant producing fungi. Other fungal species also have been reported for antioxidant production as *Cladosporium tenuissimum* [250], *Neosartorya fischeri JS0553* [253], *Lasiodiplodia venezuelensis* [255], and *Pseudopestalotiopsis. camelliae-sinensis* [208].

### Anticancer activity

Cancer is a collection of disorders that result in the uncontrolled and abnormal proliferation of many types of cells, resulting in an abnormal cell mass [264]. Cancer is caused by both extrinsic (tobacco, alcohol, smoking, unhealthy diet, lifestyle, and external conditions such as Ultra violet or ionizing and non-ionizing radiation exposure) and intrinsic (ageing, DNA mutation, hormonal disturbance, and a compromised immune system) factors that cause the activation or inactivation of specific genes, resulting in abnormal cell growth [265]. Cancer is a major cause of death worldwide, with an increasing number of cases being reported annually. Recent advancements in cancer treatment involve the discovery and development of new and improved chemotherapeutics derived from natural sources [266]. Recent studies suggest that natural bioactive compounds isolated from endophytes may serve as alternate sources for the discovery of new anticancer drugs.

In last recent years, Aspergilli are the most abundant genus among other genera of fungal endophyttes, where studied reported that Aspergilli have promising anticancer activity [267]. The endophytic *Aspergillus* TRL1 which isolated from *Tabebuia rosea* was used to produce pulchranin as anticancer compound, where exhibited effective inhibition against human tumor cells like liver (Hep-G2) and breast (MCF-7) cell lines [52]. Moreover, new pyrano xanthones as anticancer compounds were separated from *Aspergillus* ASCLA which have anticancer activity toward human cervix carcinoma [53]. He et al. [54] reported that, Gartryprostatins A, B and C compounds which separated from endophytic Aspergillus sp. GZWMJZ-258 have anticancer activity against human FLT3-ITD mutant AML.The endophytic fungus, *A. candidus* LDJ-5 which isolatedfrom*Rhizophora apiculata Blume*, it possess prenylterphenyllins as anticancer compounds [212]. Liu et al. [213] isolated *A. flavus* from *Tylophora ovate* which has anticancer activity against breast cancer. *Aspergillus terreus and A. flavus* an endophytic fungi were isolated from *Ficus elastica* have potential anticancer activity against MCF7, LS174 T, HCT29, HEPG-2 cell lines [214]. Tawfike et al. [215] separated 5-hydroxymellein, diorcinol, botryoisocoumarin A and mellein from endophytic *A. flocculus* wich exhibited anticancer activity against K562 cancerous cell line. Aspermicrones A-C compounds were separated from *A. micronesiensis*, where have anticancer activity against HepG2 cell line [216]. A. neoniger produced asperpyrone D and Aurasperone D which have anticancer activity against human immortal erythroleukemia cells [218]. Endophytic Penicilli have been reported as antioxidant producers. The endophytic *P. ochrochloronthe* which isolated from Taxus media have anticancer activity against five tumor cell lines (A549, LN229, MGC, LOVO, and MDA231) [181]. Ming et al. [57] separated xanthones compounds from *Penicillium vsp. ct-28 which had efficacy* on cell cycle and apoptosis in human hepatoma HepG2 cells. Palanichamy et al. [226] separated anticancer compounds alterchothecenes and trichothecenes from endophytic *Alternaria* sp. sb23 which applied on colon and breast cancer. Wang et al. [225] separated anticancer compounds includes dibenzo-α-pyrone derivatives, a benzo-γ-pyrone derivative and an amide-type compounds from endophytic *A. alternata,* where these compounds have anticancer activity against human cancer cell line A-549, MDA-MB-231, MCF-7, KB and KB-VIN.

Flavipin is an anticancer compound isolated from endophytic C. globosum, where has anticancer activity against cancer cell lines A549 and HT-29 [268]. Furthermore, pretrichodermamide G, a novel epidithiodi ketopiperazine (ETP) as bioactive compounds were isolated from endophytic fungus *Trichoderma harzianum* and* Epicoccum nigrum* [234]*.* Another recent studies reported that, *Diaporthe eres* [236], *Nigrospora sphaerica* [200], Phoma *bellidis* [244],* P. macrostoma* [245],* Emericella sp. TJ29* [246] and *Curvularia sp.* [247] have anticancer activity against common human cancers. Moreover, un common fungi such as *Chaetosphaeronema hispidulum* [249], *Bipolaris sp. L1–2* [251], *Myrothecium roridum spp.* [252], *Paraphaeosphaeria sp. F03* [206], *Hypomontagnella Monticulosa* [254], *Phomopsis sp.* [257], *Phyllosticta capitalensis* [209], *Paraphaeosphaeria sp. F03* [206], *Pestalotiopsis sp.* [258] have been reported for anticancer activity.

**Fig. 2:**
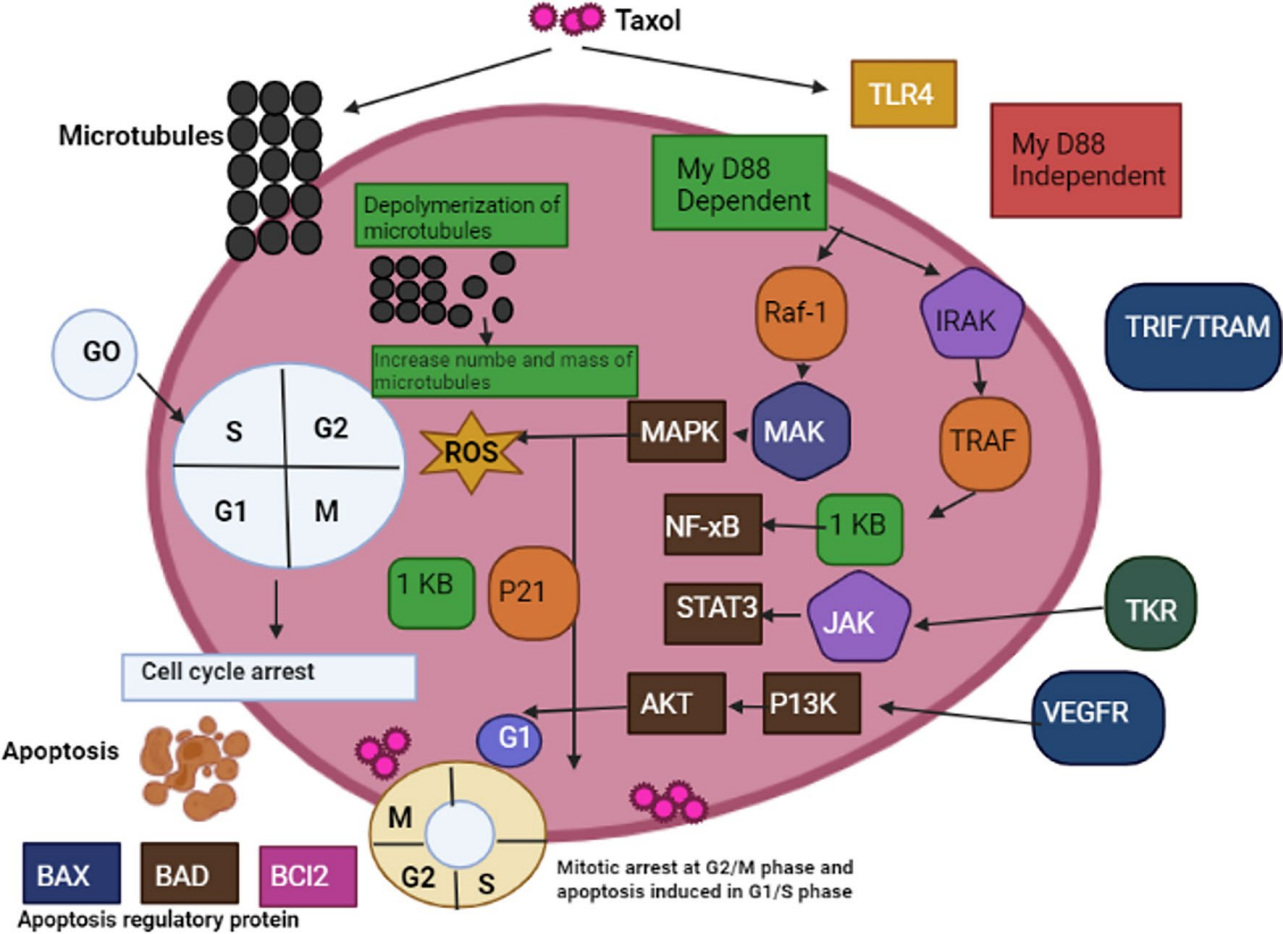
Mechanism of action of Taxol as anticancer

Taxol is the common anticancer bioactive compound which produced from fungal endophytes. Taxol is a diterpenoid isolated from yew tree of *Taxus brevifolia* which is wildly used as anticancer compound [269]. Mechanism of action of taxol was described in Fig. 2. Taxol prevents depolymerization of microtubules, where microtubules interfers to assembly of mitotic spindle and separation of chromosomes, which this leads to mitotic arrest of cell and caused the death [270]. Also, taxol has antiangiogensis activity, where helps in suppressing the expression of vascular endothelial growth factor (VEGF) of breast cancer [271]. Moreover, taxol is disturbed microtubule network, arrested G2/M, increased Bax/Bcl-2 ratio, these lead to apoptosis and damage of tumor cell [272]. Furthermore, taxol is activated TLR4, this promotes the the initiation and mobilization of Lyg6C + and Lyg6G + myeloid progenitor cells into tumors. Also, activation of TLR4 leads to denovo generation of intratumoural lymphatic vessels that were extremely lenient to attack the malignant cell.

## Conclusions

Currently, we are losing the fight against ineffective, toxic, and expensive therapeutic antimicrobial drugs. Over the past few years, endophytic fungi have concerned great attention in the drug advance process as they are ubiquitous and abundantly availability. Endophytic fungi are microorganisms that thrive in the epidermis and aerial sections of plants, which contain a vast array of chemical compounds. Endophytic fungi produce biologically active secondary metabolites, such as terpenes, alkaloids, monoterpenoids, peptides, and polyketides. Fungal endophytes are used to control a wide range of human health issues, such as numerous microbial pathogens, such as fungi, bacteria, and viruses, through various mechanisms, such as the production of antibiotics, antifungal, antiviral, anticancer, lytic enzyme, and degradation of toxins. Endophytic fungi have metabolic variety and own unique secondary metabolite pathways, which will pave the way to different metabolite isolation and applications in medicine. This review reported that some biological activities of endophytic fungi constitute an important source of biologically active substances of medical importance, and the extracts of these isolates were characterized by their antioxidant activity, which encourages research on biologically active secondary receptors that would solve many health problems in humanity. Thus, we concluded application of fungal endophytes for synthesis of bioactive compounds against microbial diseases instead of chemicals. Also expand the application of the fungal endophytes and develop the methods of formulating so that the application is safer, easy, cheap and more effective. It must be taken into account, the existence of an untapped microbial wealth that produces a lot of new bioactive compounds within the inner cells towards biotechnological progress to accelerate the screening of new biomolecules to treat many life-threatening diseases, thus preserving human health, which ensures the discovery of active compounds. Novel biologics for potential applications in the medical and pharmaceutical industries.

## Data Availability

The datasets generated during and/or analyzed during the current study are available from the corresponding author on reasonable request.
