# Correction to: Bioactive compounds and biomedical applications of endophytic fungi: a recent review

**DOI:** 10.1186/s12934-023-02131-0

**Published:** 2023-07-05

**Authors:** Amr H. Hashem, Mohamed S. Attia, Eslam K. Kandil, Mahmoud M. Fawzi, Ahmed S. Abdelrahman, Mohamed S. Khader, Mohamed A. Khodaira, Abdallah E. Emam, Mohamed A. Goma, Amer M. Abdelaziz

**Affiliations:** Botany and Microbiology Department, Faculty of Science, Al-Azhar University, Cairo, Egypt

**Correction to: Microbial Cell Factories (2023) 22:107**

10.1186/s12934-023-02118-x

In the original publication of the article [1], the author’s name Eslam K. Kandil was incorrectly written as Esalm K. Kandil. The original article has been corrected.
